# Evaluation of a Registered Nurse‐Led Outpatient Paracentesis Service for Decompensated Cirrhosis: A Retrospective Review

**DOI:** 10.1111/jep.70578

**Published:** 2026-09-08

**Authors:** Molly McDonald, Carmen Fang, Christine Song, Colina Yim, Pam Hubley, Ina Cherepaha‐Kantorovich, Sharlene Camaya, Elizabeth Lee

**Affiliations:** ^1^ Toronto Centre for Liver Disease, Toronto General Hospital University Health Network Toronto Ontario Canada; ^2^ Collaborative Academic Practice University Health Network Toronto Ontario Canada

**Keywords:** ambulatory care, cirrhosis, paracentesis, registered nurse

## Abstract

**Background:**

Ascites is a common and debilitating complication of decompensated cirrhosis that frequently requires therapeutic paracentesis (TP) for symptom relief. Limited outpatient procedural capacity may contribute to avoidable hospital readmissions. The use of registered nurses (RNs) to perform TP remains uncommon, with few models described internationally.

**Objective:**

To evaluate the safety of an RN‐led outpatient TP model and describe the potential geographic reach of patients accessing care at a Canadian tertiary liver clinic.

**Methods:**

A retrospective chart review was conducted of all outpatient TP procedures performed at Toronto General Hospital between 6 June 2022 and 28 February 2023. Procedures were performed either in the Medical Day Unit (MDU; performed by physicians/nurse practitioners) or in the Liver Clinic (LC) procedure room by trained RNs. The primary outcome was the occurrence of predefined procedure‐related adverse events. Adverse events were identified through review of clinical documentation, including emergency department visits within the institutional health network and patient‐reported events documented in the electronic medical record. A secondary analysis examined geographic patterns of access using patient residential postal codes.

**Results:**

A total of 124 patients underwent 580 TP procedures, of which 55.2% were performed by RNs. Alcohol‐associated liver disease and metabolic dysfunction‐associated steatotic liver disease were the most common underlying etiologies. No procedure‐related complications were identified (0%; 95% CI: 0–0.52%). Most patients resided within the Greater Toronto Area; however, some travelled up to 360 km, often bypassing closer hospitals, to access outpatient services.

**Conclusions:**

An RN–led outpatient TP model demonstrated reassuring safety signals and contributed to expanded procedural capacity within an ambulatory liver programme. These findings support the feasibility of nurse‐led procedural models as a strategy to improve access to care for patients with decompensated cirrhosis. Further evaluation is needed to assess the impact of this approach on healthcare utilisation and patient‐reported outcomes.

## 1 Background

Cirrhosis represents the most advanced stage of chronic liver disease and is an increasing global health burden [1, 2]. In Canada, it is among the leading causes of hospital readmissions [3]. Over the past two decades, the incidence of cirrhosis has nearly doubled, now driven primarily by metabolic dysfunction‐associated steatotic liver disease (MASLD) and alcohol‐associated liver disease (ALD) [1, 2]. Complications of cirrhosis, including hepatic encephalopathy, variceal bleeding and ascites, are associated with significant morbidity and mortality and often require complex medical management in the ambulatory and inpatient settings. Thirty‐day readmission rates exceed 30% [4, 5, 6].

Ascites is a common and debilitating complication of cirrhosis that contributes to significant symptom burden, including pain, dyspnoea, sleep disturbances, fatigue, nausea and vomiting [7]. Therapeutic paracentesis (TP), a bedside medical procedure used to remove ascites from the abdominal cavity using a needle or catheter, is essential for symptom relief. Large‐volume paracentesis is typically defined as removal of 5 litres or more of ascitic fluid [8]. For the purposes of this study, TP refers to the removal of any volume of ascites. Although diuretics remain first‐line pharmacologic therapy for ascites management, many patients are unable to tolerate them due to electrolyte imbalances and renal dysfunction. Definitive interventions, such as trans‐jugular intrahepatic portosystemic shunt (TIPS) insertion may not be feasible in some patients due to clinical contraindication [9, 10].

Hospital readmissions for reaccumulating ascites account for approximately 24.7%–32.3% of unplanned hospital readmissions for decompensated cirrhosis [4, 11]. Many of these admissions may be avoidable with timely access to outpatient TP appointments. However, access to ambulatory TP appointments is frequently constrained due to limited procedural capacity and competing clinical demands [7, 12]. Innovative models of care are urgently needed to improve timely access to outpatient TP and reduce preventable hospitalisations, given the increased global healthcare workforce shortage and rising prevalence of cirrhosis [2, 13].

The Toronto Centre for Liver Disease (TCLD) Liver Clinic (LC), based at Toronto General Hospital (TGH) at the University Health Network (UHN), is the largest ambulatory liver care clinic in Canada and serves a high volume of patients with decompensated cirrhosis. Prior to 2018, outpatient TPs were performed by physicians (MDs) and nurse practitioners (NPs) in the Medical Day Unit (MDU). Despite advance scheduling, increasing demand resulted in persistent Emergency Department (ED) and General Internal Medicine (GIM) utilisation for urgent TPs. Patients also frequently presented to clinic visits in decompensated states requiring immediate intervention, further straining available resources.

In response to these capacity constraints, the LC introduced a dedicated procedure room in 2018 staffed by a trained registered nurse (RN) to perform TPs, aiming to expand outpatient capacity for TP access and reduce avoidable readmissions beyond the Medical Day Unit (MDU). In Ontario, TP is a controlled act that RNs may perform under the direct order from an authorised provider (NP or MD), in accordance with the College of Nurses of Ontario and within the RN's scope of practice (SOP) [14]. To our knowledge, this represents one of the first reported RN‐led outpatient paracentesis models in North America, with limited comparable programmes described internationally (UK and New Zealand) [15, 16]. The limited adoption of such models may reflect variability in SOP interpretation, access to training, and institutional support for expanding nursing roles. Contemporary nursing frameworks emphasise that scope of practice is not defined by specific tasks, but by knowledge, skills, and clinical judgement required to deliver safe and effective care, and is expected to evolve in response to population health needs and healthcare system demands [17].

Appointments in both the MDU and LC were scheduled based on capacity rather than clinical acuity. The LC procedure room is staffed only by trained RNs. Therefore, TPs in the LC procedure room are performed by an RN. In contrast, TPs in the MDU are performed only by MDs or NPs. All TPs were performed under ultrasound guidance. In both the LC procedure room and MDU, RNs also administer intravenous (IV) albumin during the same visit to reduce the risk of paracentesis‐induced circulatory dysfunction, typically using one 100 mL bottle of 25% albumin for every 3 liters of ascites drained, in accordance with institutional protocols. Standard post‐procedural care included application of skin adhesive and dressing, patient education regarding wound care, and monitoring of vital signs before and after the procedure. Patients continued to receive concurrent medical management from hepatology MDs and NPs, allowing for real‐time clinical decision‐making.

To support the RN‐led paracentesis model, a structured, competency‐based training curriculum was implemented, integrating both cognitive and psychomotor components. Trainees first completed a standardised didactic session covering cirrhosis and ascites pathophysiology, abdominal ultrasound interpretation, procedural steps, complication management, and escalation protocols. Clinical psychomotor development followed a graduated pathway, beginning with structured observations of expert proceduralists and transitioning to directly supervised performance. Practical competency required a minimum of three observed and three supervised procedures, with additional cases completed as necessary to ensure technical proficiency. Final competency assessment comprised real‐time evaluation via a standardised procedural checklist, an 80% minimum passing score on a written safety exam, and formal verification by the supervising proceduralist prior to independent practice.

### 1.1 Study Purpose

The use of RNs to perform TP remains underutilized globally, and there is limited awareness of the full scope of RN practice across healthcare organisations. This study aimed to formally evaluate the safety profile of an RN‐led paracentesis model. A secondary objective was to examine the geographic reach of the Liver Clinic (LC) and Medical Day Unit (MDU) in providing outpatient TP for patients with decompensated cirrhosis.

## 2 Methods and Data Collection

This study protocol was approved by the Research Ethics Board (REB) at the University Health Network (UHN).

A retrospective chart review was conducted to address the primary and secondary study objectives using clinical data from UHN's Electronic Medical Record (EMR). Data were extracted and stored into a password‐protected database with multi‐factor authentication, accessible only to members of the study team. The study period spanned from 6 June 2022 (the launch of UHN's new EMR system), to 28 February 2023. The dataset included all patients (18 years or older) with decompensated liver disease who underwent outpatient TP in either the Medical Day Unit (MDU) or Liver Clinic (LC) procedure room at Toronto General Hospital (TGH). All patients were followed at UHN by a hepatologist or a hepatology‐specialised NP who served as the primary provider responsible for liver‐related care. Patients with peritoneal drains for ascites management were excluded.

### 2.1 Primary Objective

A retrospective chart review was conducted to identify procedure‐related adverse events following outpatient TP performed in the Medical Day Unit (MDU) and Liver Clinic (LC) procedure room [18, 19, 20].

The following adverse events were predefined based on commonly reported complications in the literature:

1. Skin Infection
2. Bowel perforation
3. Abdominal wall abscess
4. Haematoma/Haemorrhage
5. Arterial injury
6. Catheter fragment left in the abdominal wall/cavity

These specific outcomes were selected through team consensus informed by prior literature [18, 19, 20]. Spontaneous bacterial peritonitis (SBP) was not classified as a procedure‐specific adverse event, as it is, by definition, a spontaneous complication of cirrhosis rather than a direct consequence of paracentesis.

The unit of analysis for safety outcomes was each individual TP procedure. To identify potential complications, each patient's EMR was reviewed from the date of their initial procedure through the end of the study period. Progress notes, procedure notes, and admission and discharge summaries from all encounters within the patient's UHN record, were examined for evidence of complications. Patient‐reported events were included if documented in the EMR by a UHN practitioner.

Due to REB constraints, only adverse events documented within the UHN system were captured. Complications presented to non‐UHN institutions were not systematically captured unless subsequently reported by the patient and documented in UHN EMR. No predefined observation window was applied for adverse event identification, as clinically significant complications may present at variable time points following TP. This approach was selected to maximise sensitivity for detecting complications within available data sources.

A subgroup analysis of adverse events was conducted based on proceduralist type, including registered nurses (RN), nurse practitioners (NP), or physicians (MD). Data extraction was guided by a standardised protocol that was developed, pilot‐tested, and reviewed by an independent reviewer prior to data extraction. To enhance data reliability, a second reviewer independently validated 20 patient charts, which were randomly selected using a random number generator. Of these patients' charts, 11 patients had multiple Medical Day Unit or Liver Clinic appointments for TP, and all TPs performed in the study period were reviewed. The second reviewer utilised the same methodology as the primary reviewer to identify TP‐related adverse events. This secondary review was conducted for validation purposes rather than formal inter‐rater reliability testing.

### 2.2 Secondary Objective

The secondary objective was to examine the potential geographic reach of patients accessing outpatient TP services in the Liver Clinic (LC) procedure room and Medical Day Unit (MDU). Residential postal codes of patients who underwent TP at the MDU and/or LC during the study period were collected and stored in a password‐protected database with multi‐factor authentication, accessible only to the study team. Geographic analysis was performed using aggregated postal code data to characterise regional distribution while minimising the risk of patient re‐identification. Hospital locations were identified using publicly available institutional data sources. Spatial visualisation was conducted using mapping software (Google Maps) to examine the distribution of patients relative to available hospital sites and to illustrate patterns of travel, including cases where patients may have bypassed closer institutions to access outpatient paracentesis services at UHN.

## 3 Results

### 3.1 Primary Objective

During the data collection period, 124 patients underwent at least one TP in either the Medical Day Unit (MDU) or the Liver Clinic (LC) procedure room. A total of 580 TP procedures were performed. The majority were completed in the LC (55.2%), compared to 44.8% in the MDU (Table 1). Some patients underwent procedures in both locations. The number of TPs required per patient varied (Table 2).

**TABLE 1:** Procedure location.

Procedure Location	Proceduralist	Number of TPs completed (*n* = 580)
Medical Day Unit	NP, MD	260 (44.8%)
Liver Clinic	RN	320 (55.2%)

**TABLE 2:** Therapeutic paracentesis (TP) averages.

Mean TPs per patient	4.7
Median TPs per patient	2
Range of TP procedures per patient	1–36

Alcohol‐associated liver disease and metabolic dysfunction‐associated steatotic liver disease were the primary underlying etiologies in this sample, aligning with current epidemiological trends as leading causes of cirrhosis (Table 3) [1, 2].

**TABLE 3:** Disease etiology (unit of analysis: patient).

Disease Etiology	Number of Patients (*n* = 124)
Alcohol‐Associated Liver Disease	50 (40.3%)
Metabolic Dysfunction‐Associated Steatotic Liver Disease	28 (22.6%)
Viral Hepatitis Infection	17 (13.7%)
Autoimmune Liver Disease	7 (5.6%)
Other	22 (17.7%)

Patient‐level clinical severity measures (e.g., Child–Pugh score or Model for End Stage Liver Disease score) were not collected, as the primary objective of this study was to evaluate the procedural safety of TP rather than to assess outcomes stratified by disease severity. The unit of analysis was the procedure itself, and safety outcomes were evaluated at the level of each paracentesis. However, the absence of clinical severity data limits the ability to examine whether complication risk varied by underlying disease acuity.

No procedure‐related complications were identified across all procedures, regardless of proceduralist type (RN, NP, or MD). Using a rule‐of‐three approximation, the upper bound of the 95% confidence interval for complication risk was estimated at 0.52% per procedure. Comparative analyses between groups were planned a priori; however, as no complications occurred, statistical comparisons were not performed and results are presented descriptively (Table 4).

**TABLE 4:** Procedure‐related adverse events (unit of analysis: procedure).

Adverse event	Number of occurrences (%)
Skin infection	0 (0%)
Bowel perforation	0 (0%)
Abdominal wall abscess	0 (0%)
Haematoma/Haemorrhage	0 (0%)
Arterial injury	0 (0%)
Catheter fragment left inside abdominal wall/cavity	0 (0%)

### 3.2 Secondary Objective

**FIGURE 1:**
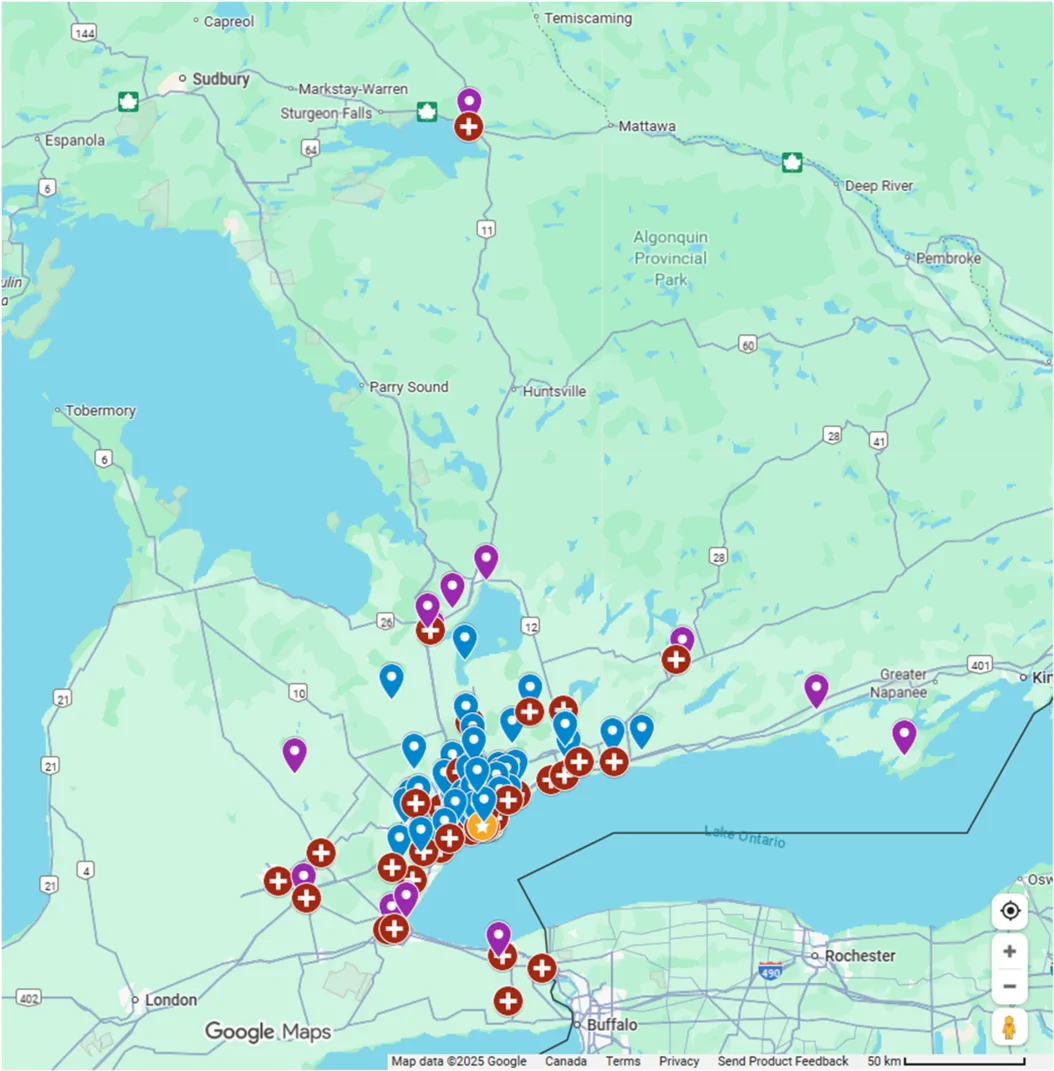
Patients requiring TP during study period and regional hospital coverage. Star: Toronto General Hospital (TGH), Red: hospitals, Blue: patients requiring TP within Toronto and GTA, Purple: patients requiring TP outside of Toronto and GTA. Data on patients' residence location was based on their postal code. All postal codes were aggregated to protect patients' identities.

Of the 124 patients included in the study, 111 resided within the City of Toronto and the Greater Toronto Area, which spans approximately 7125 square kilometres and may correspond to travel times of up to 2 h to Toronto General Hospital in either direction (Figure 1).

**FIGURE 2:**
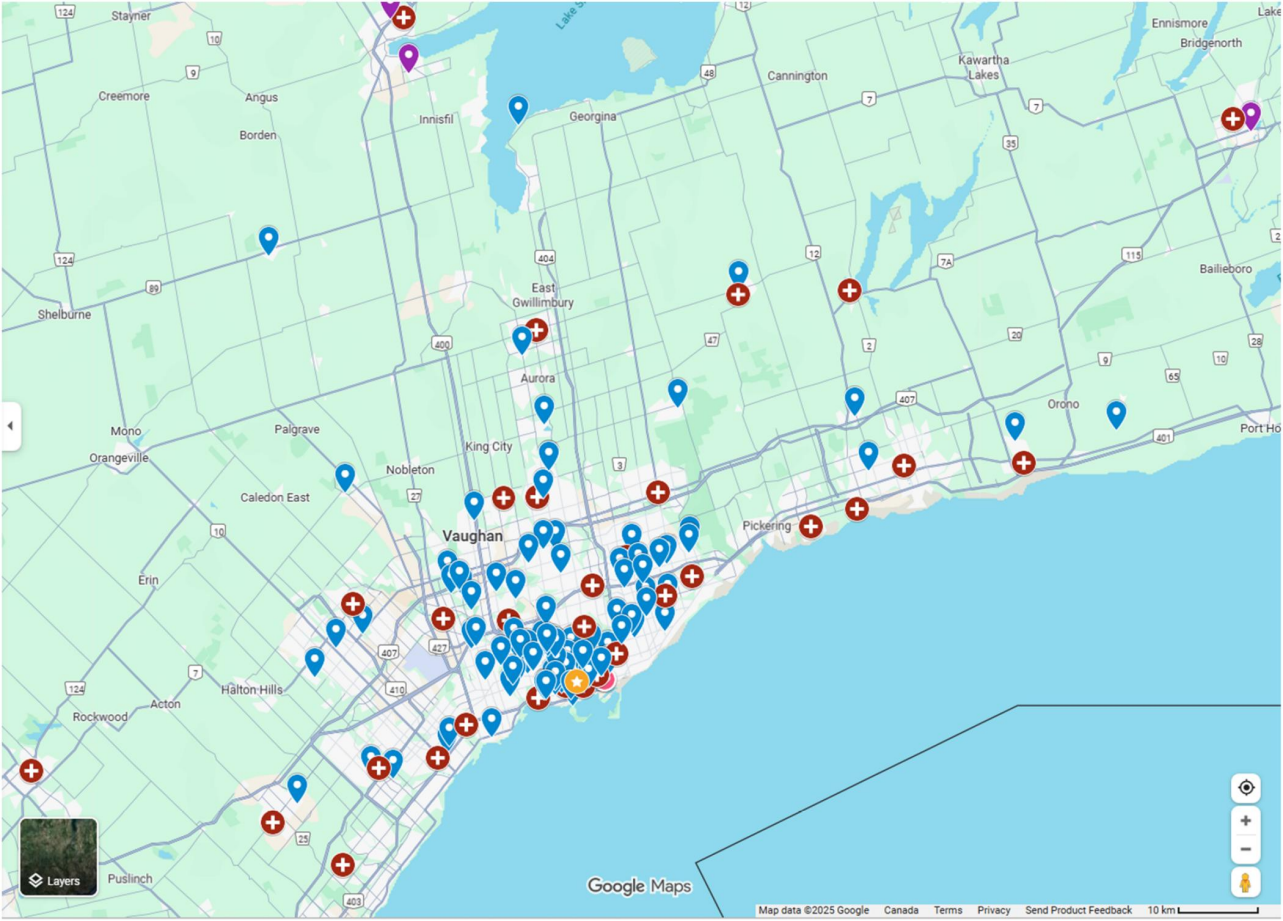
Patients requiring TP during study period: Toronto and GTA. Star: Toronto General Hospital (TGH), Red: hospitals, Blue: patients requiring TP within Toronto and GTA. Data on patients' residence location was based on their postal code. All postal codes were aggregated to protect patients' identities.

Patients travelled distances of up to 360 km to access outpatient TP services, reflecting limited availability of comparable ambulatory care locally. Within the City of Toronto and the Greater Toronto Area, there are approximately 30 academic and community hospitals, excluding paediatric and specialty centres (Figure 2). Summary measures of travel time were not calculated, as travel duration is highly variable depending on geographic region, traffic conditions, and mode of transportation. Given the heterogeneity of urban and rural travel patterns within the study population, distance‐based estimates were also not derived.

Despite this geographic distribution of hospitals, patients frequently travelled beyond closer institutions to receive care at Toronto General Hospital. These findings illustrate the potential geographic reach of outpatient TP services and suggest variability in access to ambulatory procedural care across institutions.

## 4 Discussion

### 4.1 Strengths

This study represents one of the first evaluations in North America of a RN‐led outpatient TP model and provides important insights into its feasibility and potential safety within an ambulatory liver programme. A key strength of this study is the relatively large number of procedures evaluated, with over 500 TP procedures included in the analysis. In addition, this is among the first studies to characterise the geographic distribution of patients accessing outpatient paracentesis services and to highlight gaps in access across healthcare institutions.

Two key findings emerged. First, the RN‐led model demonstrated reassuring safety signals, with no procedure‐related complications identified across all procedures, regardless of proceduralist type. Second, patients travelled considerable distances, potentially bypassing closer hospitals, to access outpatient TP, suggesting variability in the availability of ambulatory procedural services.

The absence of observed complications should be interpreted cautiously. While the findings support a low complication rate, they do not indicate that the true risk is zero. Using a rule‐of‐three approximation, the upper bound of the 95% confidence interval for complication risk was estimated at 0.52% per procedure. As such, rare but clinically meaningful adverse events cannot be excluded. These findings therefore support the feasibility and potential safety of the RN‐led model, rather than definitive equivalence across provider groups.

This study highlights the role of workforce optimisation in addressing growing demand for cirrhosis‐related care. As the prevalence of patients with MASLD‐ and ALD‐related cirrhosis rises, healthcare systems face increasing pressure to provide timely access to symptom‐relieving interventions such as TP. Enabling trained RNs to perform these procedures expands capacity without increasing reliance on physician or NP procedural time, aligning with broader health system strategies to optimise scope of practice and improve efficiency in chronic disease management.

Geographic findings further underscore system‐level access challenges, as patients frequently travelled past closer institutions to receive TP at a tertiary centre. This pattern suggests that ambulatory TP is not uniformly available across healthcare settings. Expansion of RN‐led models may help address these gaps by increasing local procedural access and reducing travel burden for patients.

### 4.2 Limitations

Several limitations should be considered. Patient‐level clinical severity measures and demographic characteristics were not collected due to research ethics constraints, limiting the ability to assess whether complication risk varied by disease acuity or patient factors. Adverse events were captured only within encounters documented at the study institution, which may have resulted in under‐ascertainment of complications presenting elsewhere. The study period was limited to 9 months and was conducted at a single centre, which may affect generalisability. In addition, differences in procedural volume between provider groups may introduce performance bias when interpreting safety comparisons, as higher procedural volume is associated with increased technical proficiency.

### 4.3 Opportunities and Considerations for Implementing an RN‐Led Paracentesis Model

The implementation of a RN–led TP model was associated with a redistribution of procedural workload within the ambulatory care setting. During the study period, RNs performed the highest volume of procedures, reducing reliance on MDs and NPs for routine TP.

Within the context of cirrhosis care, expanding access to outpatient TP may improve patient experience through potential benefits of reducing wait times, decreasing reliance on emergency and interventional radiology services, and facilitating timely symptom relief [21, 22, 23]. Continuity of care provided by consistent proceduralists may further support therapeutic relationships and individualised patient understanding [24, 25]. The model also may enable more standardised documentation and care processes, supporting adherence to clinical guidelines and ongoing quality improvement [26].

From an implementation perspective, successful adoption of an RN‐led paracentesis model requires careful consideration of local regulatory frameworks and organisational factors. TP may not be consistently recognised as within the scope of practice of RNs across institutions, reflecting variability in policy interpretation, training availability, and institutional support [27, 28, 29, 30]. Institutions will need to ensure appropriate medical oversight, establish standardised protocols for fluid drainage and albumin administration, define escalation pathways, and develop structured training programmes to support procedural competency. Addressing these factors is essential to enable scalable and sustainable role expansion within interprofessional care models.

### 4.4 Future Directions

Future research should evaluate outcomes beyond procedural safety. Prospective studies examining emergency department utilisation, hospital readmissions, and procedural wait times would help determine the broader system impact of this model. Cost‐effectiveness analyses comparing RN‐led outpatient services with hospital‐based care may further inform resource allocation decisions. Qualitative studies exploring patient and caregiver experiences will be important to assess acceptability and identify opportunities to improve care delivery. Finally, the development and evaluation of standardised training frameworks for nurses performing TP may support broader implementation across diverse healthcare settings.

## 5 Conclusion

This study provides preliminary evidence supporting the feasibility and reassuring safety profile of an RN‐led outpatient paracentesis model for decompensated cirrhosis, with RNs performing the majority of over 500 TP procedures without identified complications. The model redistributed procedural workload within the care team, potentially reducing reliance on acute care services, and is among the first to characterise geographic gaps in access to outpatient TP.

Limitations include a single‐centre design, a 9‐month data collection period, and potential performance bias from differences in procedural volume between providers. Despite these limitations, the findings support RN‐led procedural models as a scalable, resource‐efficient strategy to expand ambulatory care capacity for patients with symptomatic ascites. Future research on these procedural models should evaluate impacts on healthcare utilisation and patient‐reported outcomes.

## Funding

The authors have nothing to report.

## Conflicts of Interest

The authors declare no conflicts of interest.

## Data Availability

The data that support the findings of this study are available on request from the corresponding author. The data are not publicly available due to privacy or ethical restrictions.

## References

[1] S. B. Roberts , B. E. Hansen , S. Shin , et al., “Internal Medicine Hospitalisations and Liver Disease: A Comparative Disease Burden Analysis of a Multicentre Cohort,” Alimentary Pharmacology & Therapeutics 54, no. 5 (2021): 689–698, 10.1111/apt.16488. 34181776

[2] J. A. Flemming , M. Djerboua , P. A. Groome , C. M. Booth , and N. A. Terrault , “NAFLD and Alcohol‐Associated Liver Disease Will be Responsible for Almost All New Diagnoses of Cirrhosis in Canada by 2040,” Hepatology 74, no. 6 (2021): 3330–3344, 10.1002/hep.32032. 34174003

[3] K. Zhao , A. Shakeri , P. Graili , J. R. Guertin , W. Wong , and M. Tadrous , “Canadian Trends in Spending on Liver Hospitalizations and Transplants: 2004–2020,” Canadian Liver Journal 6, no. 4 (2023): 407–411, 10.3138/canlivj-2022-0033. 38152325 PMC10751001

[4] R. Gaspar , S. Rodrigues , M. Silva , et al., “Predictive Models of Mortality and Hospital Readmission of Patients With Decompensated Liver Cirrhosis,” Digestive and Liver Disease 51, no. 10 (2019): 1423–1429, 10.1016/j.dld.2019.03.016. 31113738

[5] S. M. Seraj , E. J. Campbell , S. K. Argyropoulos , K. Wegermann , R. T. Chung , and J. M. Richter , “Hospital Readmissions in Decompensated Cirrhotics: Factors Pointing Toward a Prevention Strategy,” World Journal of Gastroenterology 23, no. 37 (2017): 6868–6876, 10.3748/wjg.v23.i37.6868. 29085229 PMC5645619

[6] M. Gajendran , C. Umapathy , A. Perisetti , et al., “Nationwide Analysis of Incidence and Predictors of 30‐day Readmissions in Patients With Decompensated Cirrhosis,” Frontline Gastroenterology 13, no. 4 (2022): 295–302, 10.1136/flgastro-2021-101850. 35722599 PMC9186038

[7] S. S. Rogal , L. Hansen , A. Patel , et al., “AASLD Practice Guidance: Palliative Care and Symptom‐Based Management in Decompensated Cirrhosis,” Hepatology 76, no. 3 (2022): 819–853, 10.1002/hep.32378. 35103995 PMC9942270

[8] S. Verma Diagnostic and Large‐Volume Abdominal Paracentesis – UpToDate, accessed April 13, 2026, https://www.uptodate.com/contents/diagnostic-and-large-volume-abdominal-paracentesis/print.

[9] E. W. Lee , B. Eghtesad , G. Garcia‐Tsao , et al., “AASLD Practice Guidance on the Use of TIPS, Variceal Embolization, and Retrograde Transvenous Obliteration in the Management of Variceal Hemorrhage,” Hepatology 79, no. 1 (2024): 224–250, 10.1097/HEP.0000000000000530. 37390489

[10] P. Angeli , M. Bernardi , C. Villanueva , et al., “EASL Clinical Practice Guidelines for the Management of Patients With Decompensated Cirrhosis,” Journal of Hepatology 69, no. 2 (2018): 406–460, 10.1016/j.jhep.2018.03.024. 29653741

[11] M. L. Volk , R. S. Tocco , J. Bazick , M. O. Rakoski , and A. S. Lok , “Hospital Readmissions Among Patients With Decompensated Cirrhosis,” American Journal of Gastroenterology 107, no. 2 (2012): 247–252, 10.1038/ajg.2011.314. 21931378 PMC3470789

[12] S. M. Siddique , M. Lane‐Fall , M. J. McConnell , et al., “Exploring Opportunities to Prevent Cirrhosis Admissions in the Emergency Department: A Multicenter Multidisciplinary Survey,” Hepatology Communications 2, no. 3 (2018): 237–244, 10.1002/hep4.1141. 29507899 PMC5831018

[13] T. G. Evans and N. Casamitjana , “Human Resources for Health: Health Workers, The Health System's Most Valuable Resource.” in Global Health Essentials, eds. M. C. B. Raviglione , F. Tediosi , S. Villa , N. Casamitjana , and A. Plasència . (Springer International Publishing, 2023), 297–301, 10.1007/978-3-031-33851-9_45.

[14] College of Nurses of Ontario . Scope of Practice. Published online 2023, accessed September 20, 2025, https://cno.org/Assets/CNO/Documents/Standard-and-Learning/Practice-Standards/49041-scope-of-practice.pdf.

[15] M. Tan , S. Menon , and D. Black , “The Impact on Patients of a Nurse‐Led Clinical Service in Gastroenterology,” British Journal of Nursing 26, no. 13 (2017): 734–738, 10.12968/bjon.2017.26.13.734. 28704084

[16] F. Unaç and J. Sexton , “Radiology Nurse‐Led Ascites Drainage Service in a Community Hospice,” Journal of Radiology Nursing 37, no. 1 (2018): 27–29, 10.1016/j.jradnu.2017.11.005.

[17] International Council of Nurses . Scope of Nursing Practice. accessed September 13, 2025, https://www.icn.ch/sites/default/files/inline-files/B07_Scope_Nsg_Practice.pdf.

[18] P. Cervini , G. K. Hesley , R. L. Thompson , P. Sampathkumar , and J. M. Knudsen , “Incidence of Infectious Complications After an Ultrasound‐Guided Intervention,” American Journal of Roentgenology 195, no. 4 (2010): 846–850, 10.2214/AJR.09.3168. 20858808

[19] B. A. Runyon , “Paracentesis of Ascitic Fluid: A Safe Procedure,” Archives of Internal Medicine 146, no. 11 (1986): 2259, 10.1001/archinte.1986.00360230201029. 2946271

[20] A. De Gottardi , T. Thévenot , L. Spahr , et al., “Risk of Complications After Abdominal Paracentesis in Cirrhotic Patients: A Prospective Study,” Clinical Gastroenterology and Hepatology 7, no. 8 (2009): 906–909, 10.1016/j.cgh.2009.05.004. 19447197

[21] M. W. Kroneman , H. Maarse , and J. Zee , “Direct Access in Primary Care and Patient Satisfaction: A European Study,” Health Policy 76, no. 1 (2006): 72–79, 10.1016/j.healthpol.2005.05.003. 15993978

[22] J. C. Prentice , M. L. Davies , and S. D. Pizer , “Which Outpatient Wait‐Time Measures Are Related to Patient Satisfaction?,” American Journal of Medical Quality 29, no. 3 (2014): 227–235, 10.1177/1062860613494750. 23939488

[23] T. Zhang , C. Liu , and Z. Ni , “Association of Access to Healthcare With Self‐Assessed Health and Quality of Life Among Old Adults With Chronic Disease in China: Urban Versus Rural Populations,” International Journal of Environmental Research and Public Health 16, no. 14 (2019): 2592, 10.3390/ijerph16142592. 31330818 PMC6679116

[24] A. Husain , L. Barbera , D. Howell , R. Moineddin , A. Bezjak , and J. Sussman , “Advanced Lung Cancer Patients' Experience With Continuity of Care and Supportive Care Needs,” Supportive Care in Cancer: Official Journal of the Multinational Association of Supportive Care in Cancer 21, no. 5 (2013): 1351–1358, 10.1007/s00520-012-1673-7. 23274923

[25] E. A. Bayliss , J. L. Ellis , J. A. Shoup , C. Zeng , D. B. McQuillan , and J. F. Steiner , “Effect of Continuity of Care on Hospital Utilization for Seniors With Multiple Medical Conditions in an Integrated Health Care System,” Annals of Family Medicine 13, no. 2 (2015): 123–129, 10.1370/afm.1739. 25755033 PMC4369605

[26] M. Sedarous , E. Lee , A. Bishara , et al., “Improving the Quality of Paracentesis Practices in People With Advanced Cirrhosis in an Ambulatory Care Setting,” Journal of the Canadian Association of Gastroenterology 9, no. 2 (2026): 86–93, 10.1093/jcag/gwaf041. 42058693 PMC13123676

[27] M. Birks , J. Davis , J. Smithson , and R. Cant , “Registered Nurse Scope of Practice in Australia: An Integrative Review of the Literature,” Contemporary Nurse 52, no. 5 (2016): 522–543, 10.1080/10376178.2016.1238773. 27647020

[28] J. Castner , S. Grinslade , J. Guay , A. Z. Hettinger , J. Y. Seo , and L. Boris , “Registered Nurse Scope of Practice and ED Complaint‐Specific Protocols,” Journal of Emergency Nursing 39, no. 5 (2013): 467–473.e3, 10.1016/j.jen.2013.02.009. 23639417

[29] N. Oelke , D. White , J. Besner , D. Doran , L. Hall , and P. Giovannetti , “Nursing Workforce Utilization: An Examination of Facilitators and Barriers on Scope of Practice,” Nursing Leadership 21, no. 1 (2008): 58–71, 10.12927/cjnl.2008.19691. 18448891

[30] D. D'Amour , C. A. Dubois , J. Déry , et al., “Measuring Actual Scope of Nursing Practice: A New Tool for Nurse Leaders,” JONA: Journal of Nursing Administration 42, no. 5 (2012): 248–255, 10.1097/NNA.0b013e31824337f4. 22525288

